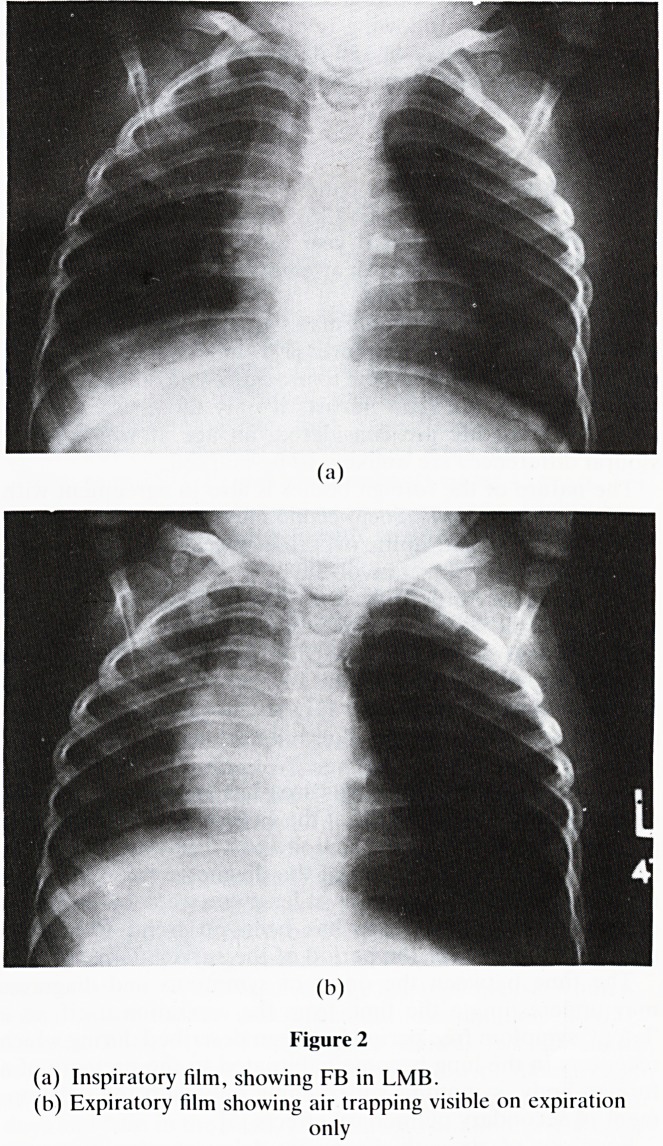# The Bristol Children's Hospital's Experience of Tracho-Bronchial Foreign Bodies 1977-1987

**Published:** 1989-08

**Authors:** AH Hamilton, F Carswell, JD Wisheart

**Affiliations:** Departments of Child Health and Cardiothoracic Surgery, Bristol Children's Hospital, St Michaels Hill, Bristol BS2 8BJ; Departments of Child Health and Cardiothoracic Surgery, Bristol Children's Hospital, St Michaels Hill, Bristol BS2 8BJ; Departments of Child Health and Cardiothoracic Surgery, Bristol Children's Hospital, St Michaels Hill, Bristol BS2 8BJ


### The Bristol Children's Hospital Experience of Tracheobronchial Foreign Bodies 1977-87

**Hamilton AH*, Carswell F, Wisheart JD***
Departments of Child Health and *Cardiothoracic Surgery, Bristol Children's Hospital, St Michaels Hill, Bristol BS2 8BJ.

### INTRODUCTION

Inhalation of a foreign body (FB) is a relatively uncommon but important cause of respiratory symptoms in childhood. In 1978 the inhalation of foreign bodies was reported to have caused 400 deaths below the age of four years in the USA [2], and an unknown number of cases of chronic pulmonary damage from persistent lobar collapse. Cases with a clear history of choking on small objects with subsequent cough and wheeze should present no diagnostic difficulty. However, it is reported that only 85% of cases have such a history, and even then it is often recalled only after the diagnosis has been made [1]. A high index of suspicion is always necessary.

The management of a case of suspected tracheobronchial foreign body in childhood has traditionally centred around bronchoscopy. In the past this was performed with a small version of the adult rigid bronchoscope; this has problems of maintenance of anaesthesia and of visibility, particularly when the grasping forceps are passed down the small lumen of the instrument [3]. These difficulties have been bypassed by technical developments in the field of endoscopy. The Storz endoscopy system utilises a rod lens arrangement with fibre-optic illumination which allows magnification of the field of view, the passage of grasping equipment under direct vision, and the maintenance of anaesthesia [3].

The purpose of this review was to evaluate the Bristol Children's Hospital (BCH) experience of tracheobronchial foreign bodies in order to assess the extent to which the management of these cases has improved during the study period, and to judge the scope for improvements in diagnosis.

### PATIENTS AND METHODS

Included in this review are those patients who have attended the BCH as primary or secondary referrals with an inhaled tracheobronchial foreign body in the years 1977 to 1987 inclusive. This includes a number of referrals from other centres comprising 19% of the total. Specifically excluded from the present study are: laryngeal or pharyngeal foreign bodies, aspiration of vomitus, aspiration of water (eg near drowning), or aspiration of a milk feed in an infant.

The patients were identified from Hospital Activity Analysis and operative records. The case notes of each child were scrutinised.

### RESULTS

In the 11 years 1977–87, 36 cases have been identified. The diagnosis was established by the finding of a foreign body in the airway at bronchoscopy (33), or by the foreign body being coughed out and identified (3). The referral rate has remained fairly constant through the review period.

The age distribution of the patients is shown in Figure 1. The second year of life (42% of patients) was the modal average; the range was 5 months to 12 years 10 months. There were 25 boys and 11 girls.

**Figure 1**

Age distribution

The nature of the foreign bodies involved is shown in Table 1. Of the total, 64% were particles of food, and 42% were peanuts.

**Table 1**

**Nature of foreign body**

| | *No* | % | |
|---|---|---|---|
| PEANUTS | 15 | 42 | 64% |
| OTHER NUTS | 4 | 11 | |
| OTHER EDIBLE | 4 | 11 | |
| PLASTIC | 5 | 14 | 36% |
| METAL | 4 | 11 | |
| GRAVEL | 2 | 5 | |
| GLASS | 1 | 3 | |
| PAPER | 1 | 3 | |

Table 2 shows the FB distribution in the lung. The intermediate group comprises those 3 in whom the foreign body was coughed out and recovered. In one case the foreign body lodged in the trachea. Otherwise, the right side predominates over the left by 50% to 39%. There is no relationship between the nature of the object and the site of deposition. In patients aged less than 2 years, the incidence of right and left sided foreign bodies is approximately equal (8 cases on R, 9 cases on L); after this age the right side predominates (10 cases vs 5) but this difference does not reach statistical significance.

**Table 2**

**Site of foreign body**

| | *No* | % | |
|---|---|---|---|
| RMB | 14 | 39 | 50% |
| RULB | 1 | 3 | |
| RMLB | 0 | 0 | |
| RLLB | 3 | 8 | |
| LMB | 12 | 33 | 39% |
| LULB | 1 | 3 | |
| LLLB | 1 | 3 | |
| TRAC | 1 | 3 | |
| INDETER | 3 | 8 | |

RMB = Right Main Bronchus
RULB = Right Upper Lobe Bronchus etc

Socio-economic groupings based on parental occupation, according to the Registrar General's classification [4], are distributed through the study group in similar proportions to the population at large.

**Presentation**

Fig 2 shows the time between the inhalation episodes and diagnosis, ranging from immediate to 120 days. No clear history of inhalation was available in 6 cases (17%), and in these instances the delay is that between the onset of symptoms and diagnosis. A group with a time lapse of 7 days or greater can be identified and defined as 'delayed'. Of these 11 patients, 6 had sought medical advice from their General Practitioner or hospital during that period without the diagnosis being made. These patients have suffered 'diagnostic delay', the remaining 5 having 'delayed presentation'. The mean delay in the early diagnosis group (25 patients) was less than 1 day; the 11 patients in the delayed group had a mean time to diagnosis of 30 days.

**Figure 2**

Distribution of time to diagnosis.

In three cases the foreign body found at bronchoscopy could not be removed, and required open thoracotomy. In each of these cases the diagnosis had been delayed at least three weeks, and all three had sought medical advice.

There is no relationship between the age of the patient and delay in presentation. Acute dyspnoea occurred in 25%, and was associated with immediate presentation. Otherwise, symptoms were relatively consistent throughout the group, with cough or wheeze being universal.

**X-rays (Figure 3)**

The majority (83%) of foreign bodies inhaled were not radio-opaque. Radio-opaque foreign bodies were diagnosed significantly earlier than those not immediately visible on x-ray ($P < 0.05$). In 22% of our cases the lung fields were clear, with or without a visible FB, 36% had loss of lung volume on the side of the deposited foreign body, and 42% had obstructive emphysema on that side. Only 3 cases were completely normal radiologically.

(a)

(b)

**Figure 2**

(a) Inspiratory film, showing FB in LMB.
(b) Expiratory film showing air trapping visible on expiration only

**Management**

Bronchoscopy under general anaesthesia is the mainstay of our management. Since 1982 this has been performed with the Storz equipment.

Bronchoscopy was carried out in all patients, and more than once in two cases. The FB was bronchoscopically removed in 30, and expelled spontaneously in 3. In 3 cases open thoracotomy was required after failure of bronchoscopy; all three cases requiring open thoracotomy occurred prior to the acquisition of the Storz system, but review of the notes suggests that the foreign body could not have been removed bronchoscopically by any means. In one, a persistently collapsed right upper lobe was found to have an organic foreign body embedded within it; another required bronchotomy for the removal of a piece of gravel which had begun to erode the bronchial wall; the third case had a plastic object wedged across the trachea at the carina.

Post-endoscopy complications occurred in 5 out of 14 cases prior to the introduction of the Storz system. These included the requirement for continuing intubation following the procedure (1 case), Dexamethasone (3 cases), repeat bronchoscopy (1), or further admission (1). There were no such complications in 22 cases endoscoped with the newer equipment. This represents a significant ($P<0.001$) improvement in the operative safety of the procedure since the introduction of the new techniques.

Long term complications were few. There were not fatalities in those reaching hospital. One patient developed persistent lobar collapse following removal of the FB; he had an initial diagnostic delay of 120 days and defaulted from long term follow up. The remainder recovered completely.

### DISCUSSION

The age distribution of the patients in this series is in broad agreement with other reviews [6]. The group most at risk is those infants in the second year of life. Parents should be made aware of this risk in an attempt to reduce the incidence of foreign body inhalation.

The sex ratio in favour of males is a phenomenon consistently noted in the literature [1], and is ill-understood. Behavioural differences seem to provide an incomplete explanation; the 2:1 sex ratio persists if only those cases in the second year of life are considered, an age at which behavioural differences are unlikely to be marked.

The nature of the foreign bodies is also in agreement with previous reviews [6, 7]. Socio-economic considerations do not appear to play a part in the overall incidence of inhalation or the nature of the object involved.

The frequency of left sided foreign bodies is greater than that found in adults, especially below the age of two years. This is a phenomenon that has been attributed to a more downward angulation of the left main bronchus from the trachea in infancy [1]; the horizontal posture of a small child might also be expected to reduce the right sided predominance.

The management of these cases has improved over the review period, both in terms of the ease of the bronchoscopic procedure for the operator, and the post-operative complication rate. The establishment of the diagnosis was difficult in many cases. In 31% there was at least one week between the event and diagnosis; there has been no trend towards a reduction in this over the period of the survey.

The time between the onset of symptoms and diagnosis may underestimate the time from the aspiration itself, as a 'latent' symptom free period has been described during which receptors in the lung become habituated to the pressure of a foreign body, symptoms supervening only with the development of secondary pulmonary effects [5].

Four out of six with diagnostic delay eventually gave a history of choking or inhalation on specific questioning after diagnosis but there was no indication that such a history had been sought initially.

In cases of doubt, radiological imaging of the chest may help confirm the need for bronchoscopy. It must be recalled, however, that few foreign bodies will be directly visible; nonetheless the effects of an object lodged in a bronchus may be seen. Lobar collapse is likely if complete obstruction has ensued; if partial obstruction has resulted in a ball-valve effect, then obstructive emphysema may occur. Obstructive emphysema may be evident on a plain chest film. In more doubtful patients it should be sought by means of comparison of expiratory and inspiratory chest films (fig 3); in young children whose cooperation is limited, lateral decubitus films or fluoroscopic screening may be helpful. However, these procedures were performed in only 11 (31%) of our cases, so the incidence of ball-valve obstruction is likely to have been underestimated. In many cases the diagnosis was clear without such techniques, but greater use of such radiological manoeuvres should improve the pick-up of obstructive emphysema and lead to earlier diagnosis. None of the 6 defined as having experienced diagnostic delay had any of these investigations. Radionuclide scanning has also been advocated to detect areas of underventilation [6]; this would only be appropriate in a minority of cases and was not employed in our series.

The diagnosis depends on clinical suspicion. A history suggestive of aspiration should be actively sought in all cases of unexplained respiratory symptoms, particularly in the second year of life. In cases where the diagnosis of inhaled FB is possible, a request for a radiological procedure to detect obstructive emphysema should improve diagnostic accuracy. The good operative safety of modern bronchoscopic techniques should encourage early referral in cases of doubt.

### SUMMARY

In the 11 years 1977 to 1987, 36 confirmed cases of tracheobronchial foreign body were seen in the Bristol Children's Hospital. The distribution of ages, sites, and natures of the objects inhaled are similar to those in other reviews.

Complicatons of removal of the foreign body once diagnosed were few, and have been absent since the Storz rod lens system was introduced in 1982.

Eleven patients experienced delay of at least 7 days between the beginning of the symptoms and diagnosis, of whom 3 subsequently required open thoracotomy. Six of these had sought medical advice during this period, of whom 4 had a positive history of choking or inhalation.

Inhaled foreign bodies remain a source of diagnostic difficulty, although this should be improved by greater attention to history and appropriate investigation. Technical advances have ensured their safe bronchoscopic removal in most cases.

### REFERENCES

1. ROTHMANN, B. F. and BOECKMAN, C. R. (1980) Foreign bodies in the larynx and tracheobronchial tree in children. A review of 225 cases. *Ann. Otol.* **89** 434–436.
2. KOSLOSKE A. M. (1980) Tracheobronchial foreign bodies in children: back to the bronchoscope and a balloon. *Pediatr.* **66**(2), 321–323.
3. ASHMORE, P. G. (1980) Foreign bodies in the bronchus in infants and children. In: Oesophageal and other thoracic problems. Wright Bristol. WILLIAMS, W. G. and SMITH, R. E., Eds. pp 138–146.
4. LITTLEJOHN, J. (1972) Studies in Sociology (6): Social Stratification. Allen & Unwin London.
5. BLAZER, S., NAVEH, Y. and FRIEDMAN A. (1980) Foreign body in the airway. *Am. J. Dis. Child.* **134**, 68–71.
6. MANTEL, K. and BUTENANT, I. (1986) Tracheobronchial foreign body aspiration in childhood — a report on 224 cases. *Eur. J. Pediatr.* **145**, 211–216.
7. COHEN S. R., LEWIS, G. B., HERBERT, W. I. and GELLER, K. A. (1980) Foreign bodies in the airway. Five year retrospective study with special reference to management. *Ann. Otol.* **89**, 437–442.

## Figures and Tables

**Figure 1 f1:**
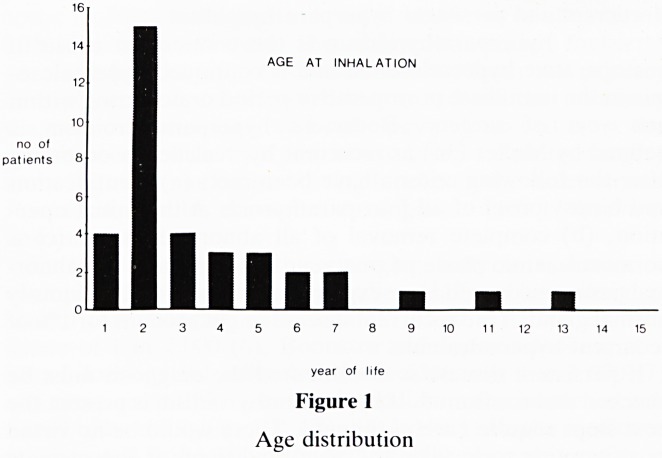


**Figure 2 f2:**
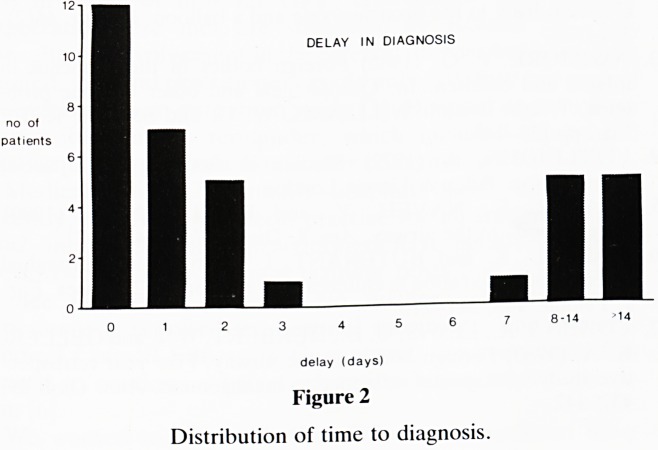


**Figure 2 f3:**